# Precast Assembled Road Paving Technology: Progress and Prospects

**DOI:** 10.3390/ma17102245

**Published:** 2024-05-10

**Authors:** Qiqi Tan, Hongzhou Zhu, Song Yang, Xiaosi Yang, Li Ou

**Affiliations:** 1 School of Civil Engineering, Chongqing Jiaotong University, Chongqing 400074, China; tanqiqi1997@126.com (Q.T.); yangsong307@126.com (S.Y.); yangxiaosi1@126.com (X.Y.); ouliyx@126.com (L.O.); 2 National and Local Joint Engineering Research Center for Transportation and Civil Engineering Materials, Chongqing Jiaotong University, Chongqing 400074, China

**Keywords:** precast assembly technology, road paving, precast cement concrete pavement, precast carpeted flexible pavement, structural characteristic

## Abstract

This article presents a systematic review of the most cutting-edge research on precast pavement technology for the first time. Firstly, precast pavement is divided into two categories, precast cement concrete pavement and precast carpeted flexible pavement, according to the application of precast technology in pavement engineering. Subsequently, the structural characteristics, advantages, and disadvantages of various precast pavement systems are compared and analyzed; technical problems in precast pavement systems are explained; and future development directions are identified. In addition, the text specifically mentions the great contribution of precast carpeted flexible pavement technology in reducing the harmful effects of asphalt fumes on humans and the environment. This work will promote the application of prefabrication in road engineering and provide suggestions and references for subsequent research.

## 1. Introduction

As a crucial development direction for engineering technology innovation and modernization in the construction field, assembly construction is a new construction method that can realize the sustainable development of green building products, save resources, and maximize the comprehensive benefits of the entire life cycle. In the field of basic transportation, which is the foundation of national economies, countries are actively conforming to the development trend of industrialization and modernization of infrastructure construction and vigorously developing and applying assembly construction technology [1]. Assembled bridges have gradually gained high positions in domestic and international bridge engineering circles, with the advantages of few traffic delays and low environmental impacts during the bridge construction process. Most small and medium-sized prestressed concrete bridges use precast assembly technology to improve the bridge quality and optimize the bridge construction period, and this technology has become one of the core techniques for the industrialization of bridge engineering production [2,3].

Compared to the application of precast assembly technology in bridge engineering, the development of precast paving technology in road engineering has been slow [4]. While overcoming the drawbacks of sloppy production, low efficiency, and difficulty in controlling construction quality caused by the traditional on-site pouring of pavement, precast pavement technology facilitates the standardized construction and refined management of pavement structures [5,6]. Furthermore, the utilization of precast pavement techniques for digital infrastructure development permits precise installation of intelligent sensing equipment within the pavement structure, leading to a considerable enhancement in the equipment’s survival rate [7]. Currently, road pavements are generally classified as cement concrete or asphalt concrete pavements; therefore, domestic and international research on precast assembled pavements has mainly focused on precast cement concrete pavement (PCCP), which is mainly composed of cement-based materials, and precast carpeted flexible pavement (PCFP), which primarily comprises flexible materials, such as asphalt-based materials or polyurethane (PU) [8,9].

In PCCP, prestressed cement concrete is mainly used, i.e., cement concrete panels are precast in a factory and then transported to the construction site for assembly and jointing. The advantages of this method are efficient laying and accurate maintenance. However, unlike continuous cast-in-place concrete pavements, precast concrete pavements are formed through the continuous splicing of pavement panels. Once spliced, the precast pavement unavoidably forms pavement joints, and the treatment of the joints is one of the difficulties of this technology [10,11,12]. Early research on PCCP technology was conducted by the Soviet Union, which began to use precast hexagonal unreinforced concrete in airport paving in 1930 [13]. Subsequently, the United States and some European countries also conducted research and development on and applied PCCP. In the 1990s, the University of Texas at Austin performed a systematic study of assembled cement concrete pavement and proposed the concept of prestressed assembled cement concrete pavement [14,15]. Currently, assembled cement concrete pavements in Europe and the United States have entered the stage of market application, and mechanical techniques, such as breaking up old road panels, drilling, and leveling subgrade, have been developed [16,17,18]. Scholars at Michigan State University have embedded sensors in precast slabs that can detect horizontal and vertical movements of precast slabs. The United States technology company Integrated Roadways, in cooperation with the Colorado Department of Transportation, piloted its developed and patented smart pavement system in Colorado, which achieves real-time traffic data collection and automatic accident alerts by building high-resolution fiber-optic sensors and other sensing technologies into precast concrete slabs [19]. Japanese scholars used precast reinforced cement concrete panels and precast high-strength reinforced cement concrete panels; developed removable cotter force transfer members; realized the mechanization of the process of precasting, placing panels, grouting, etc.; and used standard panels with preburied pipes to pave snow-melting and de-icing pavements and successfully applied them in urban roads [20]. Research on and large-scale application of PCCP technology in China started late, only after 1990. The earliest theoretical research on PCCP technology was conducted by Prof. Sun Lijun of Tongji University, who established the basic theoretical system of structural stress of interlocking block paving and simultaneously proposed the structural design method of precast concrete pavement [21,22]. Currently, the research on and application of PCCP technology is relatively mature [23].

For PCFP technology, the mainstream construction method in the international arena involves the prefabrication of flexible rollable road surfaces, lifting and transportation to the construction site, and then direct laying on the existing pavement structure. Thus far, few research results have been published on PCFPs, and the latest research has been conducted in the Netherlands, Germany, and China [24]. In 2001, the concept of carpet paving was first proposed in the “Roads to the Future” project implemented by the Dutch Ministry of Transportation, and the “Rollpave” precast paving structure based on porous asphalt concrete was developed, but its durability was found to be poor by paving test roads [25,26,27]. RWTH Aachen University in Germany was also one of the first research units to study flexible carpet pavements, and it obtained more results in the design of flexible carpet asphalt pavement structures, performance enhancement, and new flexible pavement materials [28]. In 2011, China set up the project “R&D of Rapid Maintenance Technology and Equipment for Asphalt Pavements” and successfully developed a high-viscosity and high-elasticity rollable asphalt mixture that satisfies the requirements of asphalt pavement precasting in 2015 [29]. However, PCFP technology is not limited to asphalt-based materials; synthetic materials such as PU and epoxy resin can also be used. Due to its high degree of novelty and the difficulty of production and construction, PCFP technology is still in the testing phase in various countries and has not yet been widely implemented [30].

Precast pavement technology is gradually becoming a construction solution for municipal, airport, and temporary roads and is particularly suitable for the maintenance of damaged pavements owing to its rapid laying and maintenance characteristics. However, precast pavement technology has many more applications, and the development of this technology is aimed at realizing the assembly of all pavements and maintaining consistency with the function of conventional pavements. Precast pavement technology is rarely used on high-grade highways, and there are many problems involving production or construction that remain to be solved.

Reviewing the relevant literature indicates that a systematic overview of the application of precast paving technology in road engineering is lacking. Therefore, this article provides a comprehensive summary of precast pavement technologies worldwide, which is necessary for a better understanding of the subject. This paper is mainly divided into two parts about PCCP and PCFP technology. It is anticipated that this work will support the application of precast pavement technology.

## 2. Methodology

**Figure 1:**
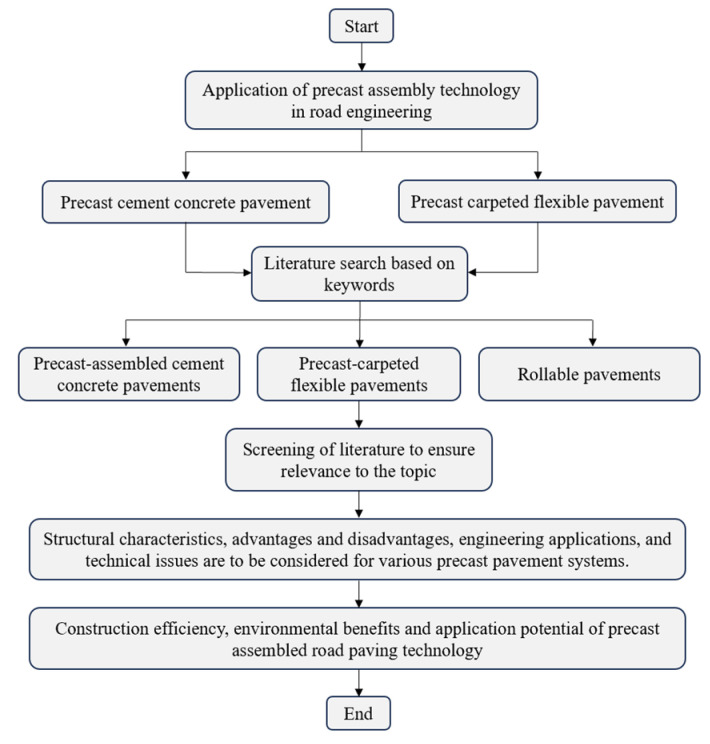
Structure of this review paper.

In this study, a systematic literature review [31] was performed to identify the applications of precast assembly technology in road engineering. This review was conducted by comprehensively and systematically collecting relevant information in several steps [32]. First, to identify the problem and topic, this paper set the overall objective of the research as the application of precast assembly technology in road pavement engineering, particularly the categorization and exploration of various types of precast pavement technologies. A literature survey was then conducted by utilizing commonly used databases, such as Web of Science, Elsevier ScienceDirect, Engineering Village, Google Scholar, Wanfang Data, and China National Knowledge Infrastructure (CNKI). Keyword searches were performed for each database, including precast assembled cement concrete pavements, precast carpeted flexible pavements, and rollable pavements. Further adjustments and screening were conducted during the literature review to ensure that the research was relevant to the topic. Finally, studies related to PCCP and PCFP technologies were categorized and reviewed. We cited 129 papers, of which about 20% were from Web of Science, about 30% from Elsevier ScienceDirect, about 12% from Engineering Village, about 15% from Google Scholar, about 8% from Wanfang Data, and about 15% from CNKI. Of the 129 papers, about 46% were published within the last five years and about 82% within the last ten years. The structure of this review paper is shown in Figure 1.

## 3. PCCP Technology

**Figure 2:**
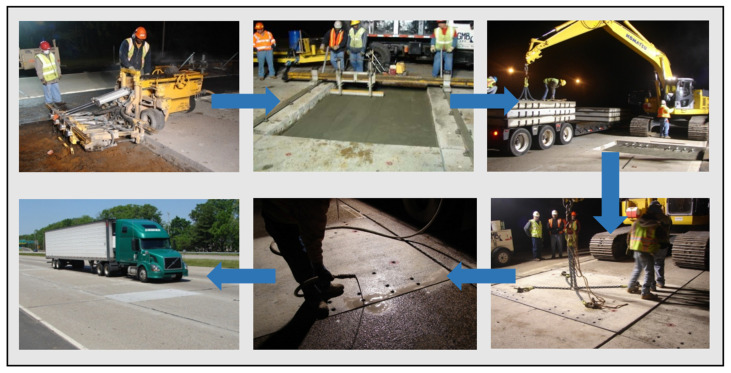
Construction of PCCP.

Precast concrete panels used in PCCP fulfill the need for rapid rehabilitation and construction of concrete pavements. Prefabricated pavement systems are manufactured in factories, transported to construction sites using transport vehicles, and installed on prepared bases (existing pavement or regraded bases) [33,34]. PCCP technology can be applied to intermittent repairs or continuous paving [35]. The main advantage of PCCP technology is that it is a truly fast, efficient, and durable pavement rehabilitation and paving technique that significantly reduces construction time and avoids traffic congestion caused by lane closures [36,37]. Precast concrete panels are used to replace damaged panels in the intermittent rehabilitation of concrete pavements. In continuous paving, precast concrete slabs can be used for reconstruction or new construction of an entire concrete pavement. The construction process of PCCP is shown in Figure 2.

### 3.1. Precast Systems for Intermittent Rehabilitation of Cement Concrete Pavements

Individual rehabilitation of concrete pavements using precast concrete panels can be performed in two types of repair: full-slab replacement of severely cracked or fragmented slabs and full-depth repair of damaged joints or cracks [38]. These repairs are full-lane width, and the construction processes for full-depth repairs and full-slab replacements are similar, except for the length of the repaired area [39]. This article describes several precast systems for the intermittent rehabilitation of concrete pavements that have been more maturely applied in solid projects.

#### 3.1.1. Super-Slab System

The Super-Slab system was developed by Fort Miller in the United States. With the Super-Slab system, pavement rehabilitation requires less than 8 h of road closure time, making it the most common precast concrete slab system and widely used worldwide [40,41]. Standard dowel bars are used to facilitate load transfer in the transverse joints of the precast panels. The ability of the manufacturer to produce slabs with different transverse gradients makes this PCCP technology suitable for the construction and repair of motorway entrance/exit ramps [42]. To date, this system has been widely utilized for production and paving. The system has been field-tested by transport authorities in several US states and Ontario, Canada [43,44].

Notably, this system is used not only for the intermittent rehabilitation of pavements but also for continuous paving operations. The pavement roughness, continuity, and performance between the pavement slabs are consistent with conventional concrete pavements, and no stress concentrations or joint damage occurs at the pavement slab joints [45]. To ensure that the slotted grout is not directly exposed to the environment or wheel loads that can cause the material to deteriorate, the dowel slots are set at the base of the Super-Slab system. However, the durability of the joints and the service life of the precast pavement are greatly influenced by the quality of the grouting [40,46].

#### 3.1.2. Stitch Slab System

**Figure 3:**
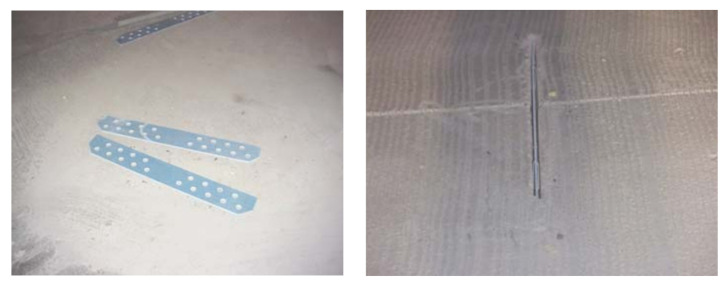
Stitch slab system [49].

In 1997, Uretek Inc. (Tomball, TX, USA) developed a stitch slab system to fix defective joints and restore load transfer to concrete pavements, which can be applied to rehabilitate single or multiple slabs [47]. Two patented techniques have been developed by Uretek. The first is the Uretek^®^ method, which utilizes high-density PU foam to lift, align, seal, and fill concrete slabs placed directly on the soil base, and the second is the Stitch-In-Time^®^ process, which is applied to restore load transfer to cracked, spalled, or otherwise damaged concrete pavements [48]. When treating pavement to be rehabilitated, it is first sealed using the Uretek method, and then load transfer is restored using the stitch-in-time process. A typical stitch slab system is shown in Figure 3. The basic features of the stitch slab system are as follows: (i) the panels are brought to the site by a haul truck and then lifted onto the treated foundation; (ii) by injecting high-density PU foam under the panels, the panels are raised to an appropriate height; and (iii) the panels are sewn to the existing slab or other panels with fiberglass sheets [49,50]. Notably, for the application of stitched panel systems with long lengths, expansion joints must be provided at intervals of 14–18 m. Otherwise, premature panel cracking may occur [51].

#### 3.1.3. Michigan Slab System

**Figure 4:**
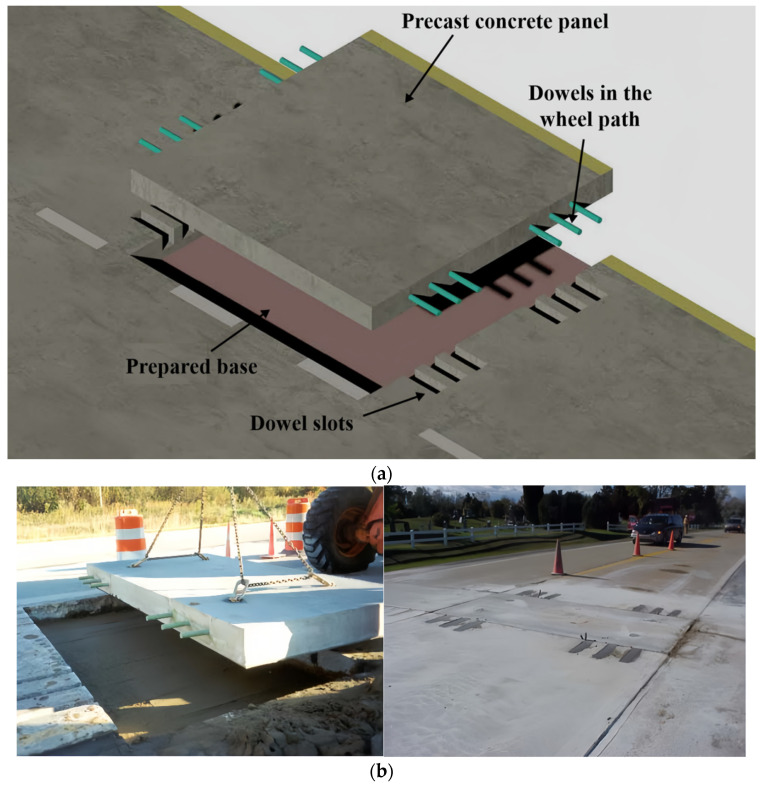
Michigan slab system [52]. (**a**) Schematic diagram. (**b**) On-site construction.

Developed by Michigan State University, the Michigan slab system is a structure characterized by precast slabs with three or four dowel bars under each wheelbase. In assembling the pavement slabs, the slabs with dowel bars are placed on top of the slabs with grooves, and the joint gaps are filled with cement-based composites [52]. Michigan’s mechanical performance is consistent with that of the Super-Slab system, and the roadway smoothness, continuity, and load transfer capacity between the roadway panels are similar to those of continuous cast-in-place concrete pavement [53]. The notches for the dowel bars are made on the top of the precast panels, making them easier to handle during the assembly phase. Figure 4 shows a schematic and a practical example of the Michigan panel system.

Typical defects exhibited by roadway panels scheduled for repair in several demonstration projects constructed in Michigan, Virginia, and Ontario include moderate to high levels of transverse cracking and spalling [54,55]. The advantages of this technique are as follows: the panels can be produced in large quantities in precast plants, depending on the geometry and proximity of the repair site; 8–10 panels can be assembled in one day; the road can be opened to traffic quickly after construction; the dowel bars at the transverse joints ensure adequate load transfer efficiency.

The key conditions required for the application of intermittent maintenance of cement concrete pavements are good support conditions under the panels, adequate load transfer capacity at the transverse joints, and minimization of the height difference between the panels and the existing pavement.

With the increasing volume of urban traffic and the urgent need to address the issue of timely maintenance of pavements, road maintenance organizations are investigating new technologies, including PCCP, to minimize construction time and make pavements more durable. Over the past decade, the research and application of PCCP technology have become increasingly sophisticated and not only technically feasible but also economically justifiable.

### 3.2. Precast Systems for Continuous Paving of Cement Concrete Pavements

Precast slabs can also be utilized for continuous paving of concrete pavements. Two types of systems are commonly used, the jointed precast concrete pavement (JPCP) system with reinforced or prestressed concrete slabs and the precast prestressed concrete pavement (PPCP) system [37,56]. Currently, a third type of continuous system has been developed and is termed an incrementally connected precast concrete pavement (ICPCP) system. The concrete slabs in this system can be either reinforced or prestressed [57].

#### 3.2.1. JPCP Systems

JPCPs resemble cast-in-place jointed concrete pavements and behave similarly after installation. They can be characterized as follows: (i) the panels are flat; (ii) the panels are reinforced with steel reinforcements; (iii) the transverse joint faces of the slabs are smooth.

**Figure 5:**
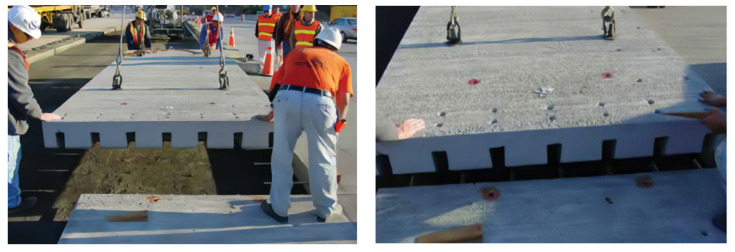
Jointed precast concrete pavement system [60].

The JPCP system used in the United States transfers loads at transverse joints. Load transfer systems must be incorporated into all JPCPs. For load transfer, JPCPs use round dowel bars (generally steel bars) [58,59]. Figure 5 provides a scheme that is utilized to effect the load transfer, which is similar in conception to the Super-Slab system. In this system, one side of the slab is provided with slots along the underside to accommodate the dowel bars, and the other side is provided with embedded dowel bars in positions to match the slots. Once installed, the slots and surrounding gaps are injected with quick-setting grout [60].

#### 3.2.2. PPCP Systems

**Figure 6:**
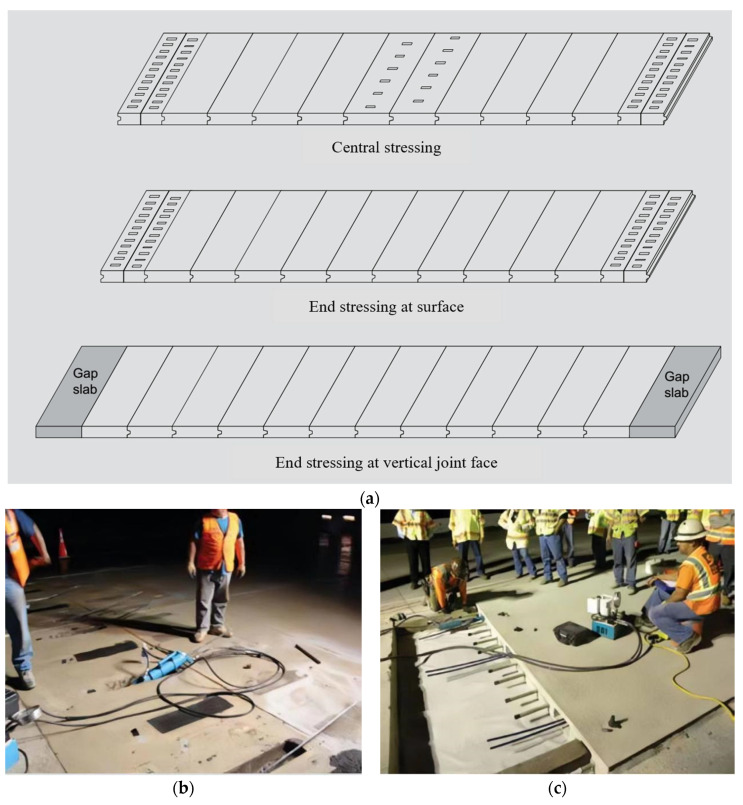
PPCP systems [65]. (**a**) Three types of PPCP systems. (**b**) Site construction of the second PPCP system. (**c**) Site construction of the third PPCP system.

PPCP systems involve the application of prestressing technology to produce pavement slabs. PPCP systems are ideally suited for continuous paving. A basic PPCP system is made up of several individual precast concrete slabs that are post-tensioned together in the longitudinal direction once installed. Each slab can also be prestressed in both the transverse and longitudinal directions. Pipes for longitudinal post-tensioning are cast into each slab during production. Post- and pretensioning counteract part of the tensile/bending stresses that occur in precast concrete slabs under traffic and environmental loads [61,62,63]. Prestressed slab systems combine the benefits of prestressed concrete and precast slabs. Using prestressing increases the dimensions of the pavement structure, decreases the thickness of the pavement and the number of precast joints, and makes the pavement smoother and more continuous [64]. There are three common types of PPCP systems, as shown in Figure 6. Figure 6b,c depict the second and third PPCP systems, respectively [65].

The use of PPCP systems has become increasingly popular in recent years because of their significant benefits, such as excellent pavement performance, cost-effectiveness, and short construction time. The features of PPCP systems include post-tensioning and compression of precast concrete slabs applied simultaneously with the longitudinal and transverse post-tensioning of steel strands. Increased prestressing helps create compressive stresses in the panel, enabling the concrete slab to behave as a thicker pavement. Previous project studies have shown that PPCP systems can improve durability and constructability and minimize traffic disruption to the public [66,67,68].

According to a report on a demonstration PPCP project constructed in the United States, the paving cost of this type of pavement is comparable to that of jointed precast concrete pavement. Properly designed and constructed PPCPs can be regarded as durable concrete pavements that require little maintenance during their service lives [69,70].

#### 3.2.3. ICPCP Systems

**Figure 7:**
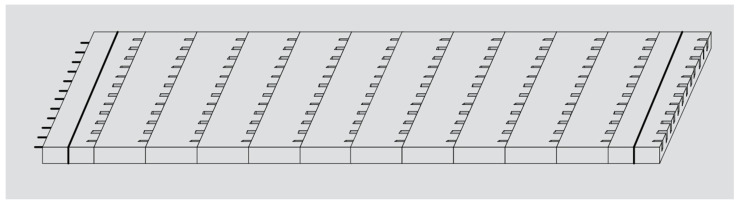
ICPCP system.

ICPCPs simulate jointed reinforced concrete pavement with hinged joints and contain panels of different lengths that are connected to allow continuous paving over long distances [71]. Deformed dowel bars, which lock the joints and ensure the necessary load transfer, are used to connect the slabs. There is a narrow expansion joint between the slabs connected [72,73]. Figure 7 illustrates an ICPCP system.

Currently, PCCP technology is relatively mature, and compared to cast-in-place cement concrete pavement, PCCP technology has the benefits of fast and efficient rehabilitation of old pavements or paving of new pavements, significantly reducing the construction time and minimizing the impact on traffic; its performance in all aspects can be made consistent with that of cast-in-place cement concrete pavement.

Based on the above analysis, a comprehensive summary of the characteristics, advantages, and disadvantages of typical PCCP systems is presented in Table 1.

### 3.3. Technical Issues to Be Considered in PCCP Systems

#### 3.3.1. Panel Reinforcement

Precast concrete panels typically use a double mat of epoxy-coated rebar bedding to minimize cracking due to lifting and transportation. Depending on the size of the panel, the amount of bi-directional reinforcement is usually at least 0.2% of the cross-sectional area of the panel. Unless the panel is designed as a reinforced concrete pavement, reinforcement is not necessary to improve pavement performance. A prominent benefit of slab reinforcement is that if the panel cracks are due to traffic loading, the cracks will remain tight without affecting the serviceability of the pavement [74,75,76].

#### 3.3.2. Production Rates

In the case of intermittent repairs within a specific lane closure, the average production rate is approximately 14–18 panels during a 6–8 h lane closure, or one panel per 20–25 min [77]. Typically, two crews are scheduled to perform construction operations: one is responsible for repairing the area, including drilling and filling the dowel bars with epoxy, and the other is responsible for installing the panels.

In continuous paving of precast concrete panels, a higher rate of panel installation can be realized, as the work is carried out over a larger area of the repair. The average production rate for slab installation is 30–40 panels for jointed systems or 122–183 m of installation length per 6–8 h lane closure [78]. For PPCP systems, the production rate may vary considerably based on the width and length of the panels. Higher production can be realized with longer slabs, as fewer slabs require assembly and are temporarily post-tensioned. Depending on the length and width of the panels, PPCP panels can be installed from approximately 61 to 183 m per 6–8 h lane closure [79,80].

In 2011, a study was carried out in the United States to determine the feasibility of rapid restoration using prefabricated techniques. For this purpose, three types of repairs were performed, namely, Repair 1 (single slab), Repair 2 (double slab), and Repair 3 [four-sided slabs (four slabs)]. The objective was to determine if the prefabricated slab repairs could be completed in a time frame of 4–6 h. The performance, speed, and cost of the prefabricated slabs were then compared to those of other repair methods. Comparisons of the precast concrete panels with those of other methods are shown in Table 2. Table 3 lists the individual installation times for each type of precast slab [81,82].

Only single-slab repair could be carried out in less than 6 h, whereas the others could be carried out in 8–10 h. As far as cost and time are concerned, the precast slab was a good option for quick pavement rehabilitation. Traditional PCC was the cheapest choice, but it did not allow for an immediate return to traffic.

#### 3.3.3. Load Transfer Efficiency

Pavement joints are important parts of rigid pavement and are the most vulnerable components in the entire pavement structure. The load transfer ability has a direct influence on the performance of the pavement structure. As jointing becomes more capable of load transfer, the pavement structure becomes more similar to a continuous panel without joints. There are three main types of joint load transfer: aggregate interlock, tongue-and-groove joints, and dowel bars [83,84].

**Figure 8:**
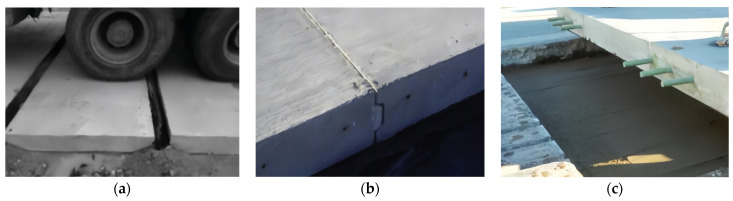
Precast pavement connection form [85,89,90]. (**a**) Aggregate interlock. (**b**) Tongue-and-groove joint. (**c**) Dowel bar.

In previous studies, aggregate interlocks have primarily been used. This joint treatment method can satisfy the sealing and waterproofing requirements of joints and realize a good load transfer function. The load transfer efficiency between slabs can exceed 60% using this connection method [85,86]. Although the aggregate interlock method is less efficient in load transfer, it is quick and easy to construct. Figure 8a shows an aggregate interlock in the field. Larrard et al. utilized hexagonal panels to pave a precast pavement, and the joints were connected using a type of aggregate interlocking with soft-pouring joints. A standard axial load of 65 kN was employed to test the pavement joints, and the vertical displacement of the joints of the pavement slabs was 1.5 mm for 5000 load cycles [87]. Liu et al. utilized prestressed panels to pave pavements with joints connected using aggregate interlocking, and the width of the slab joints was 35 mm. No defects were obvious in the pavement after one year of use. However, because of the wide joints of the pavement, the level was significantly reduced [88].

The tongue-and-groove joint method, mainly through tongue-and-groove connection, has been utilized to realize load transfer between precast panels. The tongue-and-groove acts as active hinges between the panels, and when the tongue-and-groove joints are closed, the load transfer between the panels is approximately 85% efficient. Simultaneously, the tongue-and-groove joints provide good dispersion of the maximum tensile stresses in the panels and deflections at the joints [89]. However, tongue-and-groove joints are unable to always remain in a closed working state, and once a gap is developed, the load transfer ability of the tongue-and-groove joints decreases dramatically as the joint gap becomes wider, which is caused by the characteristics of the concrete material and its working environment. Tongue-and-groove joints are shown in Figure 8b. In addition, assembling pavement panels on-site can be difficult; therefore, tongue-and-groove joints are less commonly used on precast roads.

**Figure 9:**
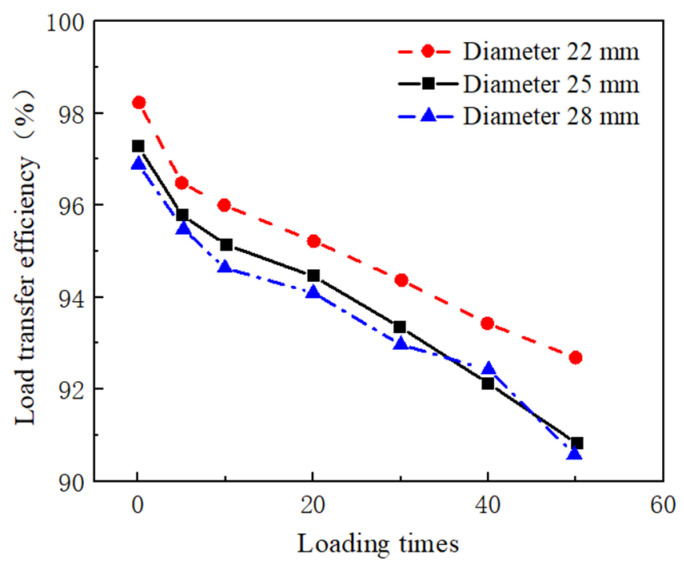
Curves of load transfer efficiency with loading times.

Currently, the most widely used connection type is the dowel bar connection, which is also considered to be the most reliable connection type, as shown in Figure 8c. The buried dowel bar method refers to the use of preburied dowel bars to connect adjacent assembled panels during the panel prefabrication process. The force transfer principle involves transferring the load by means of the shear and flexural stiffness of the dowel bars. By connecting them in this manner, the load transfer effectiveness between the panels can be as high as 95% [90]. Tayabji and Tyson studied the mechanical performance of transverse dowel bars in a Super-Slab system. The load transfer ability of the transverse joints in the Super-Slab system ranged from 85% to 95%. The joint deflection was approximately 0.2 mm under a load of 40 kN [40]. Liu experimentally analyzed the load transfer ability of cement concrete pavement joints with dowel bar connections. Figure 9 shows the variation curves of the load transfer efficiency with the number of loadings when the thickness of the slab is 22 cm and the length of the dowel bar is 35 cm. The results showed that dowel bar connections perform load transfer effectively. In terms of load transfer effectiveness under cyclic loading, the dowel bar maintains a high level of efficiency [91]. In addition, this approach provides the benefits of convenient construction and good load transfer capacity. It can also realize the preliminary flatness control requirements for the assembled pavement through the connection effect of the dowel bar. When this method is used, dowel bars are preburied on one edge of the precast panel at a length of half the length of the dowel bar and a depth of half the thickness of the panel. Simultaneously, the surface of the dowel bar is treated with an epoxy resin coating before the burial of the dowel bar to ensure that the surface of the dowel bar is smooth and corrosion resistant. The assembled pavement can be opened to traffic after grouting, smoothing, and grouting.

#### 3.3.4. Panel Support Condition

For both new construction and rehabilitation projects, pavement support is critical for long-term performance, and the support under the panels should be both firm and uniform. Larrard et al. utilized hexagonal panels to pave test pavements to provide excellent contact between the pavement panels and subgrade material. An ultrathick layer, 100 mm wide and 10 mm thick, was applied to the underside of the panel to enhance adhesion between the pavement and the subgrade [87]. Liu et al. utilized prestressed panels to pave permanent pavements with a base structure consisting of a 200 mm thick gravel layer, a 150 mm thick 5% water-stable layer, and 35 mm of coarse sand as a cushion [88].

After the construction of the precast pavement base is completed, leveling materials are needed to level the base layer for a second time. Simultaneously, the leveling material must satisfy the requirements of good flatness, ease of construction, economy of cost, and high-level early strength. The coarse sand cushion is the highest quality base-leveling material; it can be used to level the top surface of the subgrade and create an ideal working plane for surface construction while simultaneously absorbing energy and reducing the stress on the upper surface of the subgrade. Dry-mixed mortar is a common leveling material for the subgrade, which can provide important support between the pavement panels and the original roadbed and has many engineering applications owing to its low construction cost. Tang repaired damaged cement–concrete pavements using Michigan slabs. A dry-mixed mortar was applied to repair and level the damaged base. The repaired pavement subgrade was well-leveled, ensuring the accuracy and efficiency of pavement slab assembly [92]. Self-leveling mortar is a premium leveling material used for base courses. It is capable of flowing freely in the horizontal plane due to its weight, thus overcoming the difficulty of controlling the leveling of conventional cement mortar. However, self-leveling materials are unable to provide a pre-embedded space between the pavement slab and subgrade once it has been set. Self-leveling materials have few applications in engineering.

#### 3.3.5. Sealing of Pavement Joints

Researchers have developed assembly pavement joint sealing materials, such as cementitious composites, cementitious materials, asphalt concrete, rubberized asphalt sealant, quick-setting grout, MRK adhesive, and epoxy mortar. On the one hand, the selection of appropriate joint treatment materials can enhance the bonding effect between the dowel bar and the panel; on the other hand, sealing the joints prevents rainwater from penetrating the base and ensures the durability of the subgrade and joints.

Priddy et al. adopted Michigan slabs for repairing damaged pavement at airports. They filled the cutting groove of the dowel bar with quick-setting grout. The compressive strength of this filler reached 21 MPa in 3 h, ensuring efficient assembly [93]. Tayabji and Tyson used a Super-Slab system to repair damaged bus platforms. The underside of the slab was filled with quick-setting grout [40]. Syed et al. constructed a 24 m long test road using prestressed panels. Cementitious materials were used to fill the panel joints and the holes in the dowel bars [94]. Liu et al. utilized prestressed panels for permanent pavements. The indirect joints of the slabs had a width of 35 mm. The lower portion of the joint was infilled with gravel with a single particle size, and the top portion of the joint was infilled with a 30–50 mm pitch batt [88].

Although PCCP technology has made considerable progress over the past decades, many challenges remain. Table 4 lists some of the technical and institutional challenges.

## 4. PCFP Technology

PCFP is a new type of precast pavement that can be lifted and transported to a construction site and directly paved on an existing pavement structure [8]. The research and development goal of PCFP technology is to use it for paving urban roads with high traffic volumes, high-level highways, and bridge structures and for the emergency repair of damaged pavements. The performance index of the PCFP must simultaneously meet the general specification requirements, and its bending performance should satisfy high requirements.

**Figure 10:**
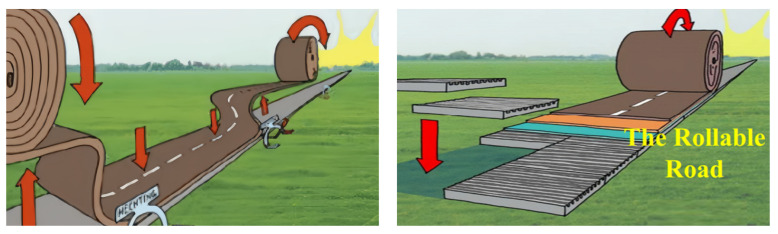
Schematic of PCFP.

PCFP technology involves four main processes: (i) prefabrication in a factory to produce an asphalt surface; (ii) curling of the precast surface on a special reel and transportation to the construction site with a special forklift truck; (iii) accurate paving on the predetermined foundation; (iv) interlayer bonding is achieved using a wireless electromagnetic wave heating reversible bonding system or by spreading tack coat. A schematic of the PCFP approach is shown in Figure 10.

### 4.1. Determination of Test Method and Evaluation Indices for the Bending Property of PCFP

**Figure 11:**
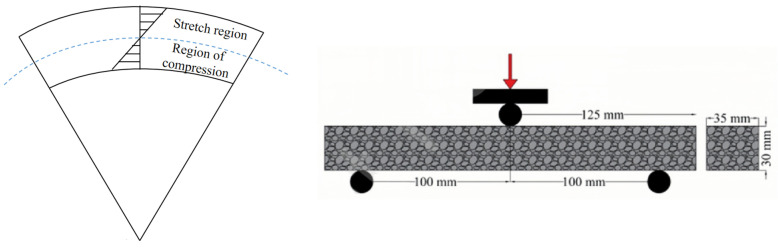
Force diagram and three-point bending test of PCFP during bending.

PCFP is characterized by the fact that the precast flexible pavement is mainly subjected to bending and tensile stresses on the underside of the pavement and compressive stresses on the top during curling, as shown in Figure 11. To ensure that the paving structure does not crack (or produce cracks that affect its strength) during curling, the bending properties of the mix should be emphasized during the mix design [95].

Assuming that the PCFP is a homogeneous elastomer, the bending strain at the underside of the pavement layer, without considering the effect of gravity, is as follows:

(1) $$\varepsilon =\frac{L_{x}-L_{z}}{L_{z}}=\frac{\frac{h}{2}}{R+\frac{h}{2}}$$

where $L_{x}$ and $L_{z}$ denote the arc lengths of the lower and neutral surfaces of the PCFP in bending, respectively; R is the radius of curvature, in mm; and h is the thickness of the pavement, in mm.

As the stress situation in which a mixture was subjected to the three-point bending test was closer to that when the PCFP was curled, a three-point bending test was applied to assess the bending performance of the PCFP. When the trabecular specimen was subjected to the three-point bending test, the bending strain at the midpoint of the bottom surface of the specimen was as follows:

(2) $$\varepsilon_{B}=\frac{6hd}{L^{2}}$$

where *L* is the sample span, in mm; h is the sample width, in mm; and d is the midspan deflection, in mm.

By combining the above two equations, the critical value of the corresponding midspan deflection of the beam in the bending test can be derived from the bending strain of the curling mix. In the bending test, if the midspan deflection at the time of damage to the beam is greater than this critical value, one can assume that the mixture does not produce cracks that affect its strength during curling [96,97,98].

Considering that PCFP is used for large traffic volumes on urban roads, high-grade highways, and bridge structures, its target reliability is 90–95%. PCFP is precast in factories, the quality of production can be guaranteed accordingly, and the overall level of variability is considered to be low. After comprehensive consideration, the reliability coefficient of the rollable mix was set to 1.2 [95]. The technical indices for the bending strain of the rollable mix are listed in Table 5. The corresponding critical values of the midspan deflections for the damage to the beams in the bending tests are listed in Table 6.

### 4.2. Research on PCFP Technology

#### 4.2.1. Asphalt-Based PCFP

**Figure 12:**
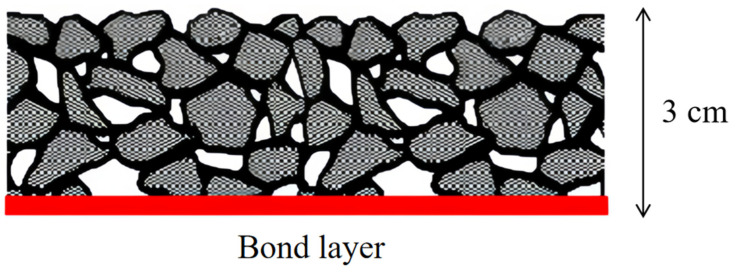
Composition of Rollpave [27].

**Figure 13:**
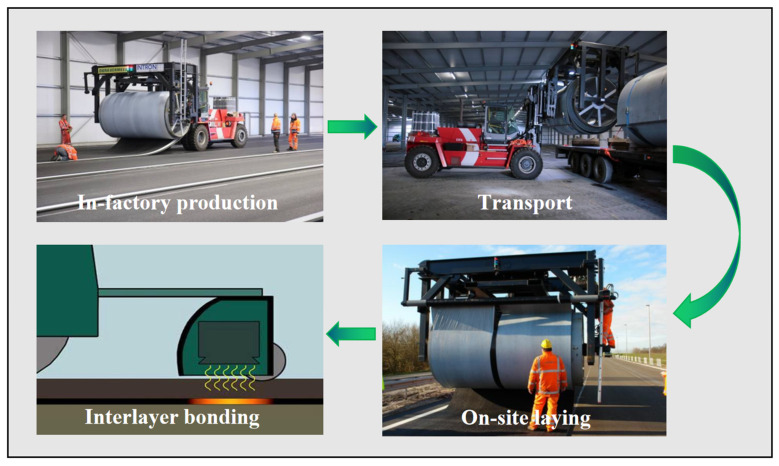
Construction process at Rollpave in the Netherlands.

In 1996, the Dutch Public Utilities and Waterworks Authority launched Roads to the Future, a research program that continues today, part of which is “modular paving technology”. Dura Vermeer-Intron, a participant in the program, developed Rollpave, a precast flexible rollable asphalt surface (thickness: 3 cm, radius of curvature: 2.5 m) that was laid and installed on site like a carpet [25]. The functional layer consists of dense-graded asphalt concrete, which effectively reduces noise and thus improves driving comfort. The bonding layer is formed by a styrene-butadiene-styrene (SBS)-modified asphalt membrane containing metallic substances and is only approximately 3 mm thick. The bonding between the Rollpave and old pavement is achieved by microwave heating (electromagnetic heating of the asphalt membrane). Figure 12 shows a schematic overview of Rollpave. The pavement enables curling because a polymer is added to the asphalt, which improves the elastic recovery coefficient of the asphalt, enhances the elasticity and abrasion resistance of the precast carpeted asphalt pavement, and reduces the probability of fracture owing to traffic, climate, and other factors. An electromagnetic induction bonding device was developed specifically for the rapid laying of Rollpave. During laying, the equipment sends out the electromagnetic field, and the steel wire mesh in the bonding layer absorbs the electromagnetic energy and converts it into heat energy to be released. Thus, the modified asphalt in the bonding layer can be melted so that the upper and lower parts of the road surface are closely bonded. The bonding process is reversible because of the presence of a metal grid in the bonding layer. When Rollpave pavements are broken and need to be repaired, electromagnetic induction equipment can be utilized to melt the bonding layer, strip the damaged Rollpave from the original pavement, and create a new pavement [26,27]. The construction process of Rollpave is illustrated in Figure 13.

Between 2001 and 2007, Dutch researchers applied Rollpave in seven practical situations. The first application was near the motorway A50, where the experimental section was 100 m × 5 m. Four Rollpave sections, each 50 m long and 2.5 m wide, were rolled onto wooden reels with an internal diameter of 2.5 m. The second application was at the Technical University of Delft, where a 20 m × 5 m test section was tested using a pavement acceleration device called LINTRACK. Considering the magnitude of the wide-base tire loads, Table 7 shows that the Rollpave test pavement outperformed the previous asphalt test sections that used only or primarily conventional asphalt mixtures. This superior performance can be attributed to the high resistance to permanent deformation of the ScorepaveM asphalt mix, i.e., very high shear strength [99].

The third application was on the A35 motorway near Hengelo, and the main purpose was to study the possibility of its large-scale application. The test section was 480 m × 12.5 m (including the fast lane, slow lane, and emergency lane). A gravel material called Neoroug was added to the test section, bringing the overall skid resistance and braking capacity of the pavement to the design requirements. However, the noise-reducing performance of the pavement did not meet the expected goals, and two years later, researchers discovered several potholes in the test section. The fourth application was in a recreational area near Deventer, with a test section of 30 m × 3 m. Traditional asphalt pavements are difficult to pave, fully demonstrating the advantages of precast pavements with low requirements for construction conditions [25]. The fifth application was on the A37 motorway near Nieuw-Amsterdam to study the feasibility of laying precast pavements at low temperatures. The test section was 430 m × 11.5 m. The results showed that the anti-skid performance of the test section did not satisfy the standard, the construction time was too long, and the noise reduction performance did not meet the requirements; however, the braking capacity satisfied the standard. Two years later, several potholes had appeared on the pavement. The sixth application was in Groningen, on an experimental section of 130 m × 3.5 m, to test the improvement of noise and anti-skid characteristics [26,27]. The seventh application was on the A37 motorway near Nieuw-Amsterdam, with a test section of 350 m × 11.5 m, to investigate whether precast pavements could be paved on curved sections. The results showed that the Rollpave could be placed on a slightly curved section with a radius of 1050 m. The construction time of this test section exceeded the expected target, and the noise-reduction performance did not satisfy the requirements. Two years later, potholes appeared on the pavement [100]. Until now, the Netherlands has not been able to make a breakthrough in this technology; therefore, Rollpave has not been popularized or applied in actual projects.

**Figure 14:**
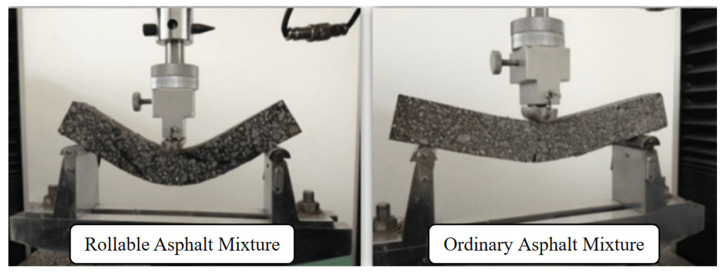
Comparative test of flexural properties of rollable asphalt mixtures and ordinary asphalt mixtures.

**Figure 15:**
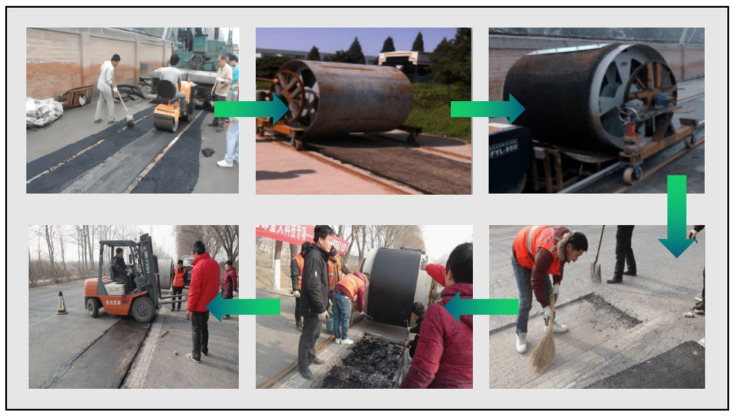
Laying process for precast rollable asphalt pavement.

Dong conducted extensive experimental research on precast rollable asphalt pavements and prepared a specialized modified asphalt binder consisting of the following materials: SK-70 asphalt, self-developed Rollpave special particles, SBS particles, furfural extraction oil, naphthenic compound oil, plasticizer A (carbon five petroleum resin), and compatibility stabilizer B (sulfur). Second, a homogeneous design method was used to optimize the existing commonly used gradation, and an optimized gradation with better bending performance was obtained. Finally, the developed rollable asphalt mixture (Figure 14) was applied to a road in Beijing, a 15 × 1.5 m pothole was cut and milled in the original pavement traveling lane, and the precast rollable asphalt pavement was laid in the pothole, as shown in Figure 15. Precast rollable asphalt pavement paving procedures are as follows: (1) remove floating dust, soil, and other pollutants from the sublayer; (2) spray modified emulsified asphalt or waterborne epoxy resin on the surface of the sublayer; (3) slowly rotate the rolls so that the pavement is laid on the sublayer; (4) fill in modified asphalt slurry in the joints; and (5) roll the paved rollable asphalt pavement two times using a small road roller. The successful paving of the test road proved that precast rollable asphalt pavements could be constructed over a large area at low temperatures. The observation results of the pavement performance of the test section indicated that its anti-skid performance satisfied the specification requirements and could be used for high-grade highways and urban roads [96,97,101]. The technical specifications of the specialized modified asphalt and rollable asphalt mixtures are shown in Table 8, Table 9, Table 10 and Table 11.

Mahdi at the Isfahan University of Technology developed a modified asphalt called Rollbit, which is a mixture of ductile agents, ethylene propylene diene monomer-modified elastomers, and bio-oil as a compounding agent. While enhancing the flexibility of the asphalt, it also enhances the rutting resistance of the asphalt mixture. In addition, to make the rollable asphalt mixtures more sustainable, some of the mineral aggregates were replaced with rubber crumbs, and their effects on the permanent deformation and bending properties were studied. The bending test results of pro-elastic hot mix asphalt (PHMA) and hot mix asphalt (HMA) are shown in Table 12 and Table 13. The maximum bending strain indicates pavement flexibility, and a higher value indicates a better resistance to cracking. It can be clearly seen that by increasing the temperature and Rollbit modified bitumen (RMB) content, the maximum bending strain and mid-depth deflection rose significantly. This is explained by the viscous behavior of bitumen at elevated temperatures; also, increasing the bitumen content can make asphalt more flexible. All Rollbit-modified HMA samples passed the rolling criteria of 5 mm mid-depth deflection at 25 °C. In contrast, just Rollbit-modified PHMA samples containing 7.5% RMB passed the rolling criteria at both 10 and 25 °C. This study was part of the research conducted by the Isfahan University of Technology on rapid and sustainable pavement maintenance to evaluate the performance of modified asphalt developed in Iran to obtain rollable asphalt mixtures [102].

Guo developed a carpeted asphalt pavement, which is mainly used for laying the upper layer of an asphalt pavement with a thickness of 3–4 cm and a maximum nominal particle size of 9.5 mm. The experimental results showed that a high dosage of SBS (12%) satisfied the curl requirements of rollable asphalt mixtures. Based on the existing dense, open-graded, and gap gradations, two types of C- and F-type gradations, which are suitable for different climatic conditions, were explored using the homogeneous design method combined with the technique of Bailey [103]. Dai developed a rollable noise-reducing asphalt pavement (with a designed thickness of 3 cm). SBS, basalt fiber, rock asphalt, and rubber powder were used to modify the asphalt matrix to obtain a special asphalt binder. A fiberglass mesh cloth was placed at the bottom of the mixture to enhance its bending capacity [97]. Shi prepared a specially modified asphalt using SBS, rubber particles, and stabilizers for an Esso 90# asphalt matrix for rollable asphalt pavements. The index requirements of the rollable asphalt mixtures were as follows: –10 °C bending and tensile strain > 8000 με, dynamic stability > 2400 times/mm, residual stability > 85%, TSR > 80%, and percolation coefficient < 120 mL/min. The test results showed that the rutting of the homemade special modified asphalt mixtures was more than 7000 times/mm, the bending and tensile strain with low-temperature bending reached up to 8400 με, the TSR reached more than 95%, the Marshall residual stability reached more than 96%, and the permeability coefficient was approximately 108 mL/min, all of which satisfied the performance requirements of precast rollable asphalt mixtures [104]. Tan innovatively developed a prefabricated flexible ultrathin overlay that can quickly and effectively repair cracks and improve skid resistance, while providing good durability. The overlay consists of basalt, SBS-modified emulsified asphalt, and warp-knitted polyester fiberglass fabric, and its unique structure makes it only one-third of the thickness of traditional ultrathin overlays. This method not only saves time and cost, but also reduces interference with road use and provides a new idea for future road rehabilitation [105].

#### 4.2.2. PU-Based PCFP

**Figure 16:**
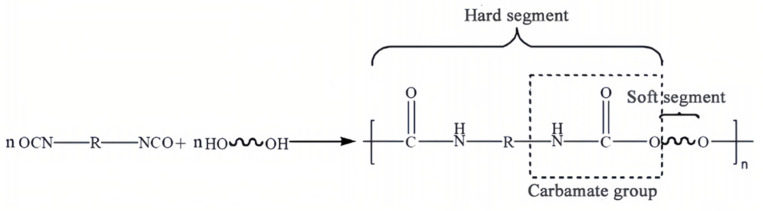
Reaction mechanism diagram for PU binders.

In contrast to China and the Netherlands, where asphalt bases are commonly used as binders for rollable mixtures, Germany has attempted to find new materials to replace asphalt. Prof. B. Steinauer of RWTH Aachen University argued that PCFP should not be limited to traditional materials and that more consideration should be given to the application of synthetic materials, resulting in carpeted pavements that are homogeneous and more suitable for industrial prefabrication [106]. Synthetic materials have garnered the attention of many researchers since 2010. Schacht and Renken conducted systematic studies based on new carpeted paving materials such as polymers and PUs. Since 2015, Renken, from RWTH Aachen University, Germany, has investigated the use of PU materials in PCFP. PU, as a polymer material, is a block copolymer made by polymerization of isocyanate and polyol, and its reaction mechanism is shown in Figure 16. The PU binder consists of hard- and soft-chain segments. The hard-chain segments are composed of polyisocyanates or their low-molecular-chain extenders, which have high glass transition and melting temperatures and provide high hardness and strength to the PU; the soft-chain segments are composed of polyester ether polyols, which make the PU pliable and elastic [19]. Therefore, the content and length of the hard- and soft-chain segments of PU binders can be artificially adjusted to achieve the desired strength and toughness.

PU is a new choice for carpeted pavements because it is stronger and more ductile than traditional asphalt and cement and can be used at both high and low temperatures. PU paving materials can achieve the required mechanical properties while simultaneously having good bending properties, and PU paving materials at room temperature can be mixed and paved in a more environmentally friendly manner. In particular, their anti-ultraviolet aging performance was significantly improved by laboratory testing after 12,000 h of ultraviolet radiation without significant changes [107].

With funding from the key project of the German Ministry of Transportation entitled “Synthesis, Preparation and Use of Synthetic Materials”, Schacht has developed a noise-reducing and anti-slip carpeted pavement made of cold plastic, which enhances the noise-reduction properties of carpeted pavements. Tests performed at the German Federal Ministry of Transportation Road Research Institute with a large drum noise testing machine showed that small and large trucks could achieve noise reductions of 6 dB and 3 dB, respectively. In addition, in a project funded by the German Ministry of Transportation, RWTH Aachen University, Germany, attempted to implant sensors with integrated functions into carpeted pavement for the first time, realizing the collection of data such as vehicle speed, traffic flow, ground pressure, temperature, humidity, and rain and snow conditions of the roadway, which in turn laid the foundation for future human–vehicle–road interactions, as well as the control and channeling of traffic flow [108].

Yang developed a precast flexible conductive composite overlay to be used for active de-icing and snow melting. The overlay is a three-layered structure with wear-resistant, functional, and heat-insulating layers from top to bottom. The wear-resistant layer is primarily composed of fine gravel with good compressive strength and anti-skid performance, which can significantly extend the service life of the overlay. Fine gravel is spread on the surface of the functional layer through an epoxy resin adhesive and using a uniform spreading method. The functional layer is made up of a PU rubber sheet in which carbon fiber heating wires are distributed and connected to an external power supply. When energized, the electrothermal properties cause the heating wire to generate heat, which, in turn, generates heat in the covering for de-icing. The insulation layer consists of an aluminum foil film, which allows more heat to transfer from the functional layer to the top and bonds to the bottom of the functional layer using a pressure-sensitive adhesive [109].

#### 4.2.3. Epoxy Resin-Based PCFP

**Figure 17:**
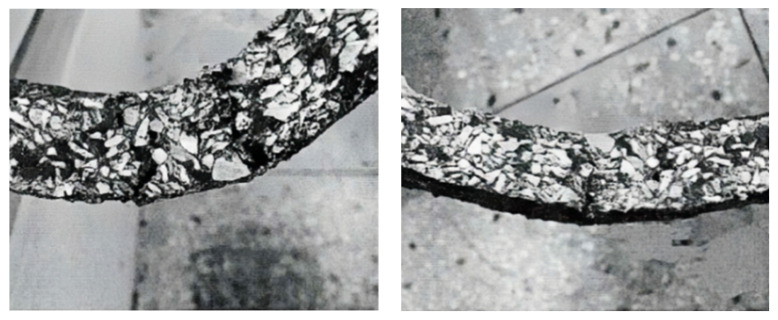
Curlable epoxy resin mix.

Le used a high-performance epoxy resin as the binder material for a mix. After utilizing softeners and tougheners, the resin was modified into a binder material with high elasticity and toughness. The grading curve of the ultrathin resin concrete was designed using the coarse aggregate void-filling method. The maximum nominal particle size was 9.5 mm, and rubber particles were added to the mixture to replace some aggregates. A polyester fiber cloth was employed as the substrate at the bottom of the mix, and a carpeted epoxy resin mix (designed thickness of 2–2.5 cm, curling radius of 1 m) was successfully prepared, as shown in Figure 17. The volume index and bending performance index were used to limit the amount of rubber particles and epoxy resin in the mixture and to determine the range; the author concluded that the amount of rubber particles should not exceed 6% and that the amount of epoxy resin should be between 12% and 15% [99].

Regarding road performance, the high-temperature performance of the epoxy resin mixture is good, and the dynamic stability is 13,000 times/mm, which is much larger than that of the carpet F-type asphalt concrete prepared by Guo. The water stability of the epoxy resin mix was comparable to that of the asphalt mix. When the dosage of epoxy resin is less than 15%, the British Pendulum Number (BPN) and texture depth satisfy the requirements of the construction standard in areas with annual rainfall greater than 1000 mm.

#### 4.2.4. Cement-Based PCFP

To deal quickly with winter damage to German pavements, Wang developed a 1 cm thick rollable pavement based on short-fiber fabric-reinforced concrete. After molding the rollable concrete, the surface was treated with epoxy resin, and crumbles with sizes in the range of 2 to 5 mm were laid down. First, a four-point bending test was used to determine the tensile strength and maximum flexibility of the rollable pavement and to compare them with the actual stresses in the real pavement. Subsequently, an Aachener raveling tester was used to polish the rollable pavement test pieces. The texture variations and their effects on acoustic properties, skid resistance, drainage, and looseness were investigated. The test results showed that this novel rollable pavement has a high level of mechanical resistance, stability, and beneficial textural properties. Using this technology, pavement damage in winter can be repaired within a few hours [110,111].

Based on the above analysis, a comprehensive summary of the characteristics, advantages, and disadvantages of the various PCFP structures is presented in Table 14.

### 4.3. Environmental Benefits of PCFP Technology

#### 4.3.1. Avoiding the Environmental and Human Health Hazards of Asphalt Fumes

In general, asphalt pavements in the field construction process involve an asphalt mixture of hot mix, paving, rolling, and other processes, and PCFP technology can maximize the simplification of the traditional asphalt pavement construction process. PCFP technology not only improves construction efficiency but also avoids the human and environmental impacts of asphalt fumes generated by conventional asphalt paving. In the construction process of tunnel asphalt pavements, large amounts of asphalt fumes will be generated and not easily removed, which will cause serious harm to the health of workers, whereas PCFP technology emits virtually no harmful substances during the construction process.

**Figure 18:**
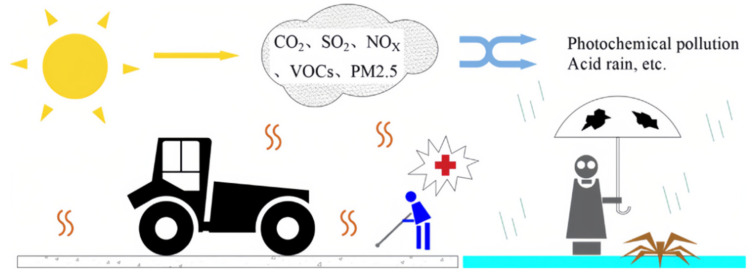
Environmental impacts of asphalt fumes from conventional asphalt pavement construction.

Asphalt fumes consist mainly of carbon oxides (CO_x_), sulfur oxides (SO_x_), nitrogen oxides (NO_x_), and volatile organic compounds (VOCs) [112]. Their harm primarily manifests as damage to the ecological environment and threats to human health. When the traditional asphalt pavement construction process is used, the asphalt smoke hazard is mainly concentrated at the paving site, as shown in Figure 18, because the majority of asphalt mixing plants have adopted effective emission control measures. Therefore, ecological environments and workers around construction sites are the most directly affected [113,114].

In China, almost 27.2 million tons of asphalt are used annually to build roads, and the emissions of CO_x_, NO_x_, SO_2_, and VOCs place a burden on the environment. Notably, VOCs are crucial precursors in the formation of ozone (O_3_) and secondary organic aerosols, and their release is the cause of the frequent occurrence of photochemical haze, which has profound effects on the production of tropospheric ozone and other oxidants [115]. Solar radiation promotes the chemical reaction of VOCs and NO_x_ to produce secondary pollutants such as peroxyacetyl nitrate (PAN) and tropospheric ozone. Photochemical smog, which is made up of acids, aldehydes, ozone, and PAN, can lead to plant mortality. Acidic chemicals in secondary pollutants also form acid rain, which poses serious threats to the structural safety and ecological health of buildings [116].

**Figure 19:**
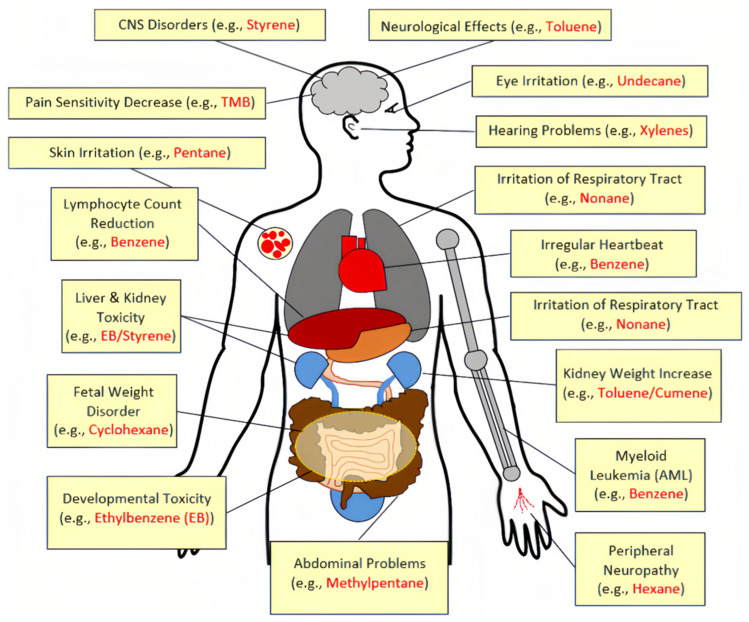
Human health hazards of VOCs [121].

Asphalt fumes are equally hazardous to humans; studies of workers exposed to asphalt fumes during asphalt paving have shown mutagenic and cytogenetic effects of asphalt fumes in humans, and experiments under controlled conditions have shown similar results [117]. Compared to the respiratory and circulatory systems, pathogenic organics entering the human body through the skin account for over 80% of all pollutants [118]. Asphalt fumes are produced and suspended in the air as aerosols. At temperatures below 110 °C, the fumes turn into a plastic glassy solid that easily adheres to the skin, destroying surface tissue and causing skin irritation or itching. The respiratory system is an important channel for the exchange of substances between the human body and the external gaseous environment. When people come into contact with asphalt smoke, it inevitably enters the body through the mouth, nose, throat, and other channels. Acridine and phenols contained in the smoke will stimulate the mucous membranes of the respiratory tract, leading to photophobia, nausea, stomach discomfort, dizziness, and other side effects. The main effects of VOCs on the human body are shown in Figure 19 [119,120,121,122,123].

#### 4.3.2. Energy Efficiency and Carbon Reduction

Highway infrastructure is not only a carrier for providing road transportation services but also an important “carbon source” for the transportation industry. Transportation accounts for 10–25% of carbon emissions in society as a whole and is the second-largest source of carbon emissions. Highways account for 75% to 85% of carbon emissions from transportation [124]. In September 2020, China explicitly proposed 2030 “carbon peak” and 2060 “carbon neutral” targets. The dual-carbon policy during the “14th Five-Year Plan” was integrated into the overall layout of ecological civilization.

**Figure 20:**
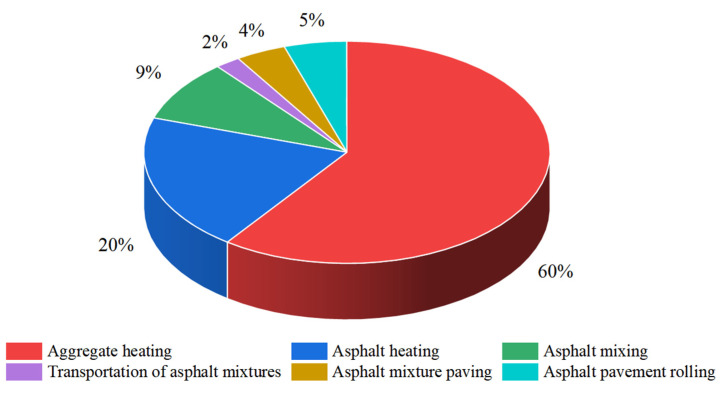
Share of carbon emissions from highways.

In the traditional asphalt surface construction process, carbon emissions arising from the mixing phase of asphalt mixtures can be divided into two aspects. First, construction machinery emissions, mixing equipment, and construction machinery in operation consume fuel and produce large amounts of greenhouse gases and other harmful gases. Second, the asphalt mixture needs to be heated to a high temperature when mixing; the higher the mixing temperature, the more energy is required, and the more carbon emissions are generated [125,126,127,128]. Figure 20 shows the percentage of carbon emissions from each aspect of the highway asphalt pavement construction process [129].

Therefore, the key to reducing the carbon emissions of asphalt pavement construction lies in decreasing the amounts of greenhouse gases, such as CO_2_, produced during the hot mixing of asphalt mixtures, as well as reducing the use of carbon-containing energy sources by construction machinery or equipment. PCFP technology avoids the traditional construction process of hot mixing of mixtures, transporting, paving, and rolling, and reduces not only CO_2_ emissions, but also the dependence on large-scale construction machinery, such as asphalt mixture dump trucks, mixers, paving machines, rollers, and other large construction machinery, which in turn decreases the consumption of fossil energy.

Traditional asphalt pavement construction is not only cumbersome and inefficient, but also affected by weather, asphalt fumes produced on the human body, and environmental hazards. PCFP technology can completely overcome the above shortcomings and is a high-efficiency, energy-saving, carbon-reducing, quality-controlled, and environmentally friendly pavement technology.

## 5. Conclusions

This article reviewed the existing literature on precast assembled road paving technologies and categorized them into PCCP and PCFP technologies based on the dominant pavement types. It compared the different types of precast pavement systems that have emerged in recent years, summarized the relevant results obtained by various countries in the field of precast pavements, and analyzed the structural characteristics, advantages, and disadvantages of various precast pavement systems. In addition, it emphasized the environmental benefits of precast pavement technology. The conclusions of the study were the following:

1. At present, the research and application of precast pavement technology is mainly based on PCCP technology, the essence of which is to improve the strength, durability, and other road performance aspects of precast cement concrete slabs to meet the needs of economic development. PCFP technology, on the other hand, has not been used on a large scale due to factors such as poor durability and technical limitations.
2. The Super-Slab and Michigan systems have a wide range of applications. PPCP systems are considered long-life concrete pavements owing to their superior durability, constructability, and cost-effectiveness and are becoming the dominant paving systems.
3. To prevent voids in assembled pavements, a secondary leveling of the spliced subgrade using a leveling material is required. Sand cushions, dry-mixed mortar, and self-leveling mortar are all premium base-leveling materials. In view of the engineering application, it is recommended to use a sand cushion for base leveling.
4. The load transfer mechanisms for assembled pavement joints are categorized into dowel bars, tongue-and-groove joints, and aggregate interlocks. Dowel bars are recommended for assembled pavements with heavy driving loads or high performance. Aggregate interlocks should be utilized for temporary paved roads or low-performance assembled pavements. Tongue-and-groove joints are to be avoided because of the limitations of the concrete material and its structural properties.
5. Considerable attention should be paid to the sealing of precast pavement joints. On the one hand, the selection of appropriate joint treatment materials can enhance the bonding effect between the dowel bar and the panel; on the other hand, sealing the joints prevents rainwater from penetrating the base and ensures the durability of the subgrade and joints.
6. PCFP technology is still quite immature, and the main problem is that durability is not guaranteed. More effectively balancing the bending of PCFP with the pavement performance is essential. The review provided in this paper demonstrates that asphalt-based and PU-based materials are still the mainstream binders for PCFP, but the poor durability of asphalt-based pavements and the high cost of PU-based pavements have always limited the popularization and application of PCFP technology. Thus, balancing the pavement performance of PCFP with cost-effectiveness is also a major challenge.

## 6. Future Work

Because of the inherent superiority of precast assembled pavement technology, precast pavement has become a popular topic in road engineering. In this paper, the application of precast assembly technology in road engineering and its effects are reviewed. The application of PCCP and PCFP has been proven to be feasible, and the research work on various aspects has been fruitful, but its potential application value has not been fully developed. Therefore, there are some recommendations for future study:

1. The authors believe that the future application of precast technology in pavement construction will still be dominated by PCCP and PCFP technology, but more advanced precast pavement technologies may exist, such as precast slab-type asphalt pavements. However, the difficulty of this technology lies in how to determine the dimensions of the slab-type asphalt pavement and how to deal with the gaps between the slabs.
2. Regarding the problem of the poor durability of PCFP technology, it is necessary to carry out a comprehensive evaluation of the road performance of PCFP and to study the interface contact behavior of PCFP structures under the action of tire loads as well as to optimize the design and improvement of carpet paving materials on this basis. The problem of a large radius of curvature of PCFP can be solved by reducing the thickness of a pavement and laying geotextiles with high tensile properties at the bottom.
3. Further mechanization and automation of precast pavement technology will be achieved using complete sets of mechanized equipment to perform operations such as original pavement breaking, substrate disposal and leveling, and precast pavement paving.
4. Precast pavement has a large development space, as a carrier, and will be integrated with increasingly advanced functions. For instance, the road surface anti-skid property, drainage, noise reduction, exhaust degradation, and energy collection and storage will be improved, which is of great significance for the current stage of road maintenance, traffic safety, and energy collection in the road area.
5. By exploring precast pavement as a substrate to promote digital road infrastructure construction, pavement can be assembled rapidly and accurately, and the deployment of sensing equipment can be standardized, thus realizing the informatization of road infrastructure. The collection of data such as vehicle speed, traffic flow, grounding pressure, temperature, humidity, and rain and snow conditions on roadways lays the foundation for future human–vehicle–road interaction and traffic flow control and diversion.

## Figures and Tables

**Table 1:** Comparison of typical PCCP systems.

System Name	Structural Feature	Advantages	Shortcomings
Super-Slab system	One end is fitted with dowel bars, the other end is fitted with notches, and the joints are connected by lower grouting	85–95% load transfer capacity	The joints of the dowel bars are connected by grouting from the bottom; the grouting quality significantly affects the durability and service life of the pavement
Stitch slab system	The post-placed dowel bar is set into the reserved groove of the original road slab or precast slab; the road surface joints are wet upper joints	Load transfer capacity up to 90%	Stress concentration still occurs at the joints due to weak dowel bars; the durability of the filling materials gradually decreases with the environment and driving load
Michigan slab system	The dowel bar is set inside the precast panels; the road surface joints are wet upper joints	Satisfactory load transfer efficiency	The durability of filled materials decreases with environmental changes and increased traffic loads
JPCP systems	The panels incorporate steel reinforcements; the transverse joint faces of the panels are smooth	No structure-related curling or warping will occur; any in-service cracks due to traffic loading remain tight	At these joints, aggregate interlock cannot be relied upon to transfer loads
PPCP systems	Large size but small thickness; lightweight	Low internal stress; high bearing capacity; good capacity of resistance deformation; reduced number of precast pavement joints	High construction costs; complex construction techniques
ICPCP systems	A narrow expansion joint exists between the connected panels	Fewer active joints and narrower expansion joints	Lack of systematic structural design and construction guidelines

**Table 2:** Comparison of precast concrete slabs with those of other methods (based on single-slab completion time).

Repair Type	Repair Detail	Time to Complete Repair	Estimated Material Cost (USD)
Rapid-setting cap/aggregate	254 mm cap 102 mm compacted aggregate	3.7 h	1375
Rapid-setting cap/foam	254 mm cap 102 mm foam	3.2 h	6040
Rapid-setting cap/injected foam	254 mm cap debris 102 mm foam	3.9 h	6020
Rapid-setting cap/flowable fill	254 mm cap 102 mm flowable fill	3.4 h	1840
Precast panel/flowable fill	279 mm panel 76 mm flowable fill	4.8 h ^a^	2500
Traditional Portland cement concrete (PCC)	356 mm PCC	28 d	540

^a^ Based on the time for completion of a single panel repair.

**Table 3:** Precast slab installation time.

Task	Time (min) ^a^		
	Single-Slab Repair	Double-Slab Repair	Quad-Slab Repair
Mark the perimeter of the distressed slab	5	10	25
Saw-cutting operations	10	30	55
Dowel slot cutting	20	55	45
Anchor drilling and installations	25	45	85
Attach crane rigging hardware	0 ^b^	0 ^b^	10
Lift distressed section	10	15	15
Dowel slot excavation (existing PCC slab)	20	65	65
Dowel slot excavation (existing PCC slab)	15	25	20
Precast panel placement	10	15	35
Compaction (if needed)	5	10	25
Removal of flowable fill from dowel slots	10	10	15
Placement of joint and dowel sealant	25	45	80
Dowel slot finishing	10	15	20
Curing	110	110	110
Total repair time (min)	275	450	605
Total repair time (h)	4.58	7.50	10.08

^a^ Times rounded to the nearest 5 min interval. ^b^ Task required less than 1 min to complete.

**Table 4:** Technical and institutional challenges for PCCP.

Serial Number	Challenges
1	Increased cost of constructing PCCP systems in the face of limited government budgets
2	Rate of installation (production) in the field
3	Optimizing various PCCP system design features
4	Ensuring the durability of PCCP systems
5	Maintaining vertical alignment at joints
6	Ready availability of nearby PCCP assembly/fabrication plants
7	Lack of adequate long-term performance history
8	Lack of best practices in the design, installation, maintenance, and repair of PCCP systems
9	No well-developed, experienced-based generic specifications on how to use PCCP
10	General lack of support for improving PCCP technologies from the precast concrete industry
11	Insufficient technical understanding of the potential of PCCP systems by highway authorities. There is a requirement for technology transfer activities in relation to the following: a. Selection criteria for the application of PCCP systems b. General specifications for the application of PCCP systems c. Design, installation, maintenance, and repair issues related to PCCP systems

**Table 5:** Technical requirements for bending strain of rollable mixes.

Thickness of PCFP/cm	Minimum Bending Strain (με) Technical Requirements for Different Radii of Curvature (mm)						
	500	625	750	875	1000	1125	1250
3	35,000	28,500	24,000	20,500	18,000	16,000	14,500
4	46,500	37,500	31,500	27,000	24,000	21,000	19,000
5	57,500	46,500	39,000	33,500	29,500	26,500	24,000

**Table 6:** Critical values of midspan deflection for damage in bending tests of rollable mixes.

Thickness of PCFP/cm	Different Radii of Curvature (mm) Correspond to the Minimum Midspan Deflection (mm) at Damage						
	500	625	750	875	1000	1125	1250
3	6.7	5.4	4.5	3.9	3.4	3.1	2.8
4	8.8	7.1	6.0	5.2	4.5	4.0	3.6
5	10.9	8.8	7.4	6.4	5.6	5.0	4.5

**Table 7:** Practical rut depth (40 °C test condition) due to standard wide-base tire loadings on main wheel track (75 kN on Rollpave test pavement, 45 kN on other test sections).

Test Section	Wheel Track ^0^	N_tot_ ^1^	Average Practical Rut Depth (mm) after N =									
			1000	2000	5000	10,000	20,000	35,000	50,000	70,000	100,000	125,000
1	V2D80L	32,750	5.2	7.1	10.3	15.1	20.3	30.1	-	-	-	-
2	V2D45L	34,600	2.8	3.1	4.3	5.1	9.5	13.3	-	-	-	-
3a	V1ZOAR MV1ZOA ^2^	20,650 21,500	1.5 1.5	1.7 2.2	4.4 3.8	6.7 6.1	10.6 9.4	-	-	-	-	-
3b	V2ZOAMR MV2ZOAM ^2^	19,711 19,675	1.5 1.5	2.1 2.1	4.6 4.2	7.2 6.4	11.3 9.3	-	-	-	-	-
4a	V1DABR	68,700	0.8	1.6	2.3	3.7	4.8	6.3	7.1	8.2	-	-
4b	V1DABR	35,500	1.2	1.7	4.2	7.8	11.4	16.5	-	-	-	-
Rollpave	-	115,500	1.0	1.4	1.4	2.7	3.6	4.4	5.1	5.5	6.2	7.4

^0^ Codes for wheel tracks on test sections 1–4b according to (3, 4, 5). ^1^ Total number of standard wide-base tire load repetitions applied on different wheel tracks. ^2^ The LINTRACK testing took place approximately 3 months after the testing of the other wheel track on the same section.

**Table 8:** Technical specifications of specialized modified asphalt.

Index	Penetration at 15 °C (0.1 mm)	Penetration at 25 °C (0.1 mm)	5 °C Ductility (mm)	30 °C Complex Modulus (kPa)	−18 °C Stiffness Change Rate (m)
Technical requirement	>30	>60	>55	<200	>0.36

**Table 9:** Recommended mineral gradation for rollable asphalt mixtures.

Sieve size (mm)	13.2	9.5	4.75	2.36	1.18	0.6	0.3	0.15	0.075
Gradation range (%)	100	90–100	40–60	26–35	20–28	16–23	13–18	10–15	8–12

**Table 10:** Ranges of design specifications for rollable asphalt mixtures.

Index	Compression Rebound Modulus (MPa)	Air Void (%)	Voids in Mineral Aggregate (%)	Voids Filled with Asphalt (%)	Asphalt–Aggregate Radio
Range	800–1200	2–3.5	15–20	79–89	6.5–8

**Table 11:** Rollable asphalt mixture flexural properties and road performance indices.

Index	Technical Requirement	Test Method
Failure stress in bending test at 10 °C (MPa)	>2.5	T 0715
Failure strain in bending test at 10 °C (με)	>27,000	T 0715
Failure strain in bending test at −10 °C (με)	>8000	T 0715
Dynamic stability (1 time/mm)	>2400	T 0719
Residual stability in immersion Marshall test (%)	>85	T 0709
Residual stability of freeze–thaw splitting test (%)	>80	T 0729
Permeability coefficient (mL/min)	<120	T 0730

**Table 12:** Bending test results of HMA and PHMA samples at 10 °C.

RMB%	HMA			PHMA		
	Maximum Force (KN)	Deflection (mm)	Maximum Bending Strain (με)	Maximum Force (KN)	Deflection (mm)	Maximum Bending Strain (με)
6.5	0.61	1.86	8370.0	N/A	N/A	N/A
7	0.60	1.94	8730.0	0.26	2.47	11,115.0
7.5	0.47	3.78	17,032.5	0.30	5.80	26,122.5

**Table 13:** Bending test results of HMA and PHMA samples at 25 °C.

RMB%	HMA			PHMA		
	Maximum Force (KN)	Deflection (mm)	Maximum Bending Strain (με)	Maximum Force (KN)	Deflection (mm)	Maximum Bending Strain (με)
6.5	0.23	6.41	28,845.0	N/A	N/A	N/A
7	0.24	7.195	32,377.5	0.13	3.79	17,055.0
7.5	0.18	11.68	52,582.5	0.18	10.23	46,035.0

**Table 14:** Comparison of various PCFP structures.

PCFP Technology	Type of Bonding Material	Advantages	Shortcomings
Rollpave technology (Netherlands, 2001–2007)	Polymer-modified asphalt	Can be applied at low temperatures; easy bonding and reversible bonding process; can be paved in slightly curved sections	Large radius of curvature; prone to potholes and poor durability
Noise-reducing and slip-resistant carpeted pavement (Germany, 2010)	Cold plastic material	Significant noise reduction	-
Precast rollable asphalt pavement (China, 2015)	Polymer-modified asphalt	Can be applied at low temperatures; small radius of curvature	High cost of specialized asphalt binders
Carpeted asphalt pavement (China, 2015)	High-dosage (12%) SBS-modified asphalt	Can be constructed at low temperatures; small radius of curvature	High cost of high-dosage SBS-modified asphalt
Carpeted pavement (Germany, 2015)	PU	Normal temperature mixing; environmental protection; ultraviolet aging resistance is significantly improved	-
Rollable pavement based on short-fiber fabric-reinforced concrete (Germany, 2016)	Short-fiber fabric-reinforced concrete	Used to repair winter damage to German pavements	-
Precast flexible conductive composite overlays (China, 2018)	Epoxy resin	Active snow melting and de-icing; small radius of curvature; very thin	Requires connection of power supply and overcurrent protection
Curlable noise-reducing asphalt pavement (China, 2019)	Composite-modified asphalt	Good noise-reduction performance	Composite-modified asphalt preparation process is complicated
Curlable epoxy wear layer (China, 2021)	High-elasticity and high-toughness epoxy resin	Small radius of curvature	High cost of bonding materials
Self-heating and self-bonding carpeted asphalt pavement (China, 2022)	SBS-modified asphalt	No need to spread tack coat on the site	The overall structure of self-heating and self-bonding carpet asphalt pavement is complex; cumbersome production technology and process
Rollable asphalt pavement (Iran, 2023)	Composite-modified asphalt	High- and low-temperature performance, viscosity, and fatigue life of modified asphalt are improved	-

## Data Availability

Data are contained within the article.
